# Numerical Analysis of Droplet Impacting on an Immiscible Liquid via Three-Phase Field Method

**DOI:** 10.3390/mi14050951

**Published:** 2023-04-27

**Authors:** Qingming Hu, Fengshi Hu, Donghui Xu, Kailiang Zhang

**Affiliations:** 1 School of Mechtranoics Engineering, Qiqihar University, Qiqihaer 161006, China; 2022908292@qqhru.edu.cn (F.H.); 15946212473@139.com (D.X.); 2 The Engineering Technology Research Center for Precision Manufacturing Equipment and Industrial Perception of Heilongjiang Province, Qiqihar University, Qiqihaer 161006, China; 3 The Collaborative Innovation Center for Intelligent Manufacturing Equipment Industrialization, Qiqihar University, Qiqihaer 161006, China; 4 College of Mechanical and Electrical Engineering, Northeast Forestry University, Harbin 150040, China

**Keywords:** droplet impacting, numerical analysis, three-phase filed method, sinking, bouncing

## Abstract

In this work, we establish a two-dimensional axisymmetric simulation model to numerically study the impacting behaviors between oil droplets and an immiscible aqueous solution based on the three-phase field method. The numerical model is established by using the commercial software of COMSOL Multiphysics first and then validated by comparing the numerical results with the previous experimental study. The simulation results show that under the impact of oil droplets, a crater will form on the surface of the aqueous solution, which firstly expands and then collapses with the transfer and dissipation of kinetic energy of this three-phase system. As for the droplet, it flattens, spreads, stretches, or immerses on the crater surface and finally achieves an equilibrium state at the gas–liquid interface after experiencing several sinking-bouncing circles. The impacting velocity, fluid density, viscosity, interfacial tension, droplet size, and the property of non-Newtonian fluids all play important roles in the impact between oil droplets and aqueous solution. The conclusions can help to cognize the mechanism of droplet impact on an immiscible fluid and provide useful guidelines for those applications concerning droplet impact.

## 1. Introduction

Microdroplets, the scale-down analytical platforms, are commonly used in the fields of analytical chemistry [1], biology [2], material synthesis [3], and so on. Understanding the impacting mechanism between a liquid-in-air droplet and an immiscible aqueous solution is important and helpful for lots of applications, such as biofabrication through inkjet printing [4,5] and droplet encapsulation [6,7,8,9,10,11,12,13] for drug delivery [14,15], PCR reaction [16,17], and preparation of Janus droplets [18] or double-emulsions [19,20]. When the oil droplet impacts with another immiscible aqueous solution, it will transiently knock a crater on the surface of the aqueous solution and lead to the floating, bouncing, jetting, or splashing of the solution [21,22,23].

As for the impacting hydrodynamics of droplets, it has accumulated the interest of researchers for many years. Now, the impacting behaviors of droplets on various receiving surfaces ranging from solid surfaces to fluidic interfaces have been studied [24,25,26]. Recently, with the advancements of high-speed imaging techniques (HSIs) and particle image velocimetry (PIV), the impacting details of droplet can be finer captured and observed [27]. Although these studies are interesting and meaningful for understanding the droplet-impacting mechanism, most of the present analyses consider a particular class of drop impact where the droplet is made of the same fluid as one of the other two phases, which essentially sets up a biphasic drop impact problem [28,29]. As for the impact between the liquid-in-air droplet and an immiscible fluid, there are a handful of reported studies that provide preliminary analyses of the impacting velocity, solution viscosity, droplet shape, and thickness of liquid film on droplet-impacting behaviors [30,31,32,33,34,35]. However, there are still some imperfect areas needing further study since the droplet-impacting behaviors are also affected by the fluid density, droplet viscosity, droplet diameter, and interfacial tensions of the three phases. In addition, these studies only focus on Newtonian fluids. In fact, there are also many non-Newtonian fluids in the fields of biology, chemistry, materials, and so on, such as human’s blood, starch solution, and mud [36,37,38,39]. Whether the non-Newtonian properties of droplet and solution affect the droplet-impacting behaviors also needs to be analyzed. Therefore, it is necessary and meaningful to systematically analyze the influences of various factors on the impacting behaviors between a liquid-in-air droplet and an immiscible fluid.

In this article, a two-dimensional axisymmetric simulation model based on the three-phase field method is established to numerically study the impacting behaviors between an oil-in-water droplet and an immiscible aqueous solution. To mimic the freefall of a droplet from a long distance without increasing the unnecessary computational time, the oil droplet with an initial downward velocity is released into the air phase first, and it migrates down under the gravity force afterward; finally, the droplet impacts with the aqueous solution when it reaches the gas–liquid interface. The effects of the fluid viscosity, density, interfacial tension, impacting velocity, droplet diameter, and the non-Newtonian properties of fluids on the impact between oil droplet and aqueous solution are thoroughly studied. The results show that the increased oil viscosity, density, interfacial tension σ_o-a_, σ_w-o_, impacting velocity, and droplet diameter will enhance the droplet penetration depth, water bouncing height, maximum water crater size, and number of sinking-bouncing circles. In contrast, the impacting effects between droplet and liquid are negatively affected by the improved water viscosity, density, interfacial tension σ_w-a_, and power-law index n_w_. The power-law index of oil n_o_ in the range of 0.7 to 2.0 has little influence on droplet impacting. These conclusions can help to know about the mechanism of droplet impact on an immiscible fluid and provide useful guidelines for those applications concerning droplet impact.

This article is organized as follows. The mathematical formulation of this problem is discussed in Section 2. The influences of fluid viscosity, density, interfacial tensions, impacting velocity, droplet diameter, and non-Newtonian properties of fluids on droplet impacting are analyzed in Section 3, before the conclusions are drawn in Section 4. The validation of the present mathematical model and necessary simulation videos are shown in the Supplementary Materials.

## 2. Mathematical Formulation

### 2.1. Problem Description

**Figure 1:**
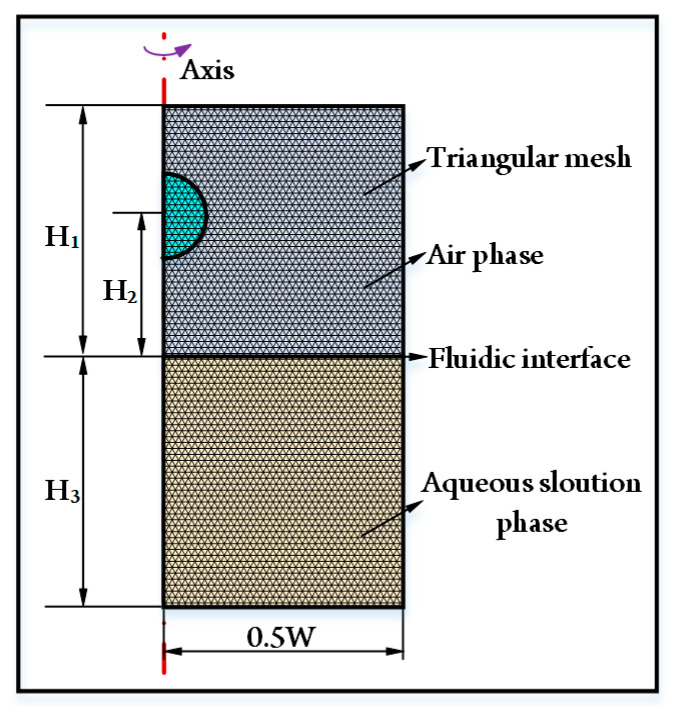
Two-dimensional axisymmetric computational domain with triangular meshes for numerical simulation. The dimensions of the computational domain are H_1_ = 20 mm, H_2_ = 10 mm, H_3_ = 20 mm, W = 40 mm, respectively.

Oil-in-air droplet impacting with an aqueous solution refers to three-phase fluids. To numerically analyze the droplet-impacting behaviors, a two-dimensional simulation model based on the three-phase field method is established by using the commercial software of COMSOL Multiphysics. The axisymmetric geometrical model with triangular meshes shown in Figure 1 is adopted. The upper computational domain with a depth of H_1_ is the air phase (*i* = *a*). The lower computational domain with a height of H_3_ is the aqueous solution phase (*i* = *w*). As for the oil droplet phase *(i* = *o*), its initial release height is H_2_. The width of the computational domain is 0.5 W. The specific values are H_1_ = 20 mm, H_2_ = 10 mm, H_3_ = 20 mm, and W = 40 mm, respectively. The droplet diameter D ranges from 4 mm to 12 mm in this study. To mimic the freefall of a droplet from a long distance without increasing the unnecessary computational time, the droplet has an initial downward velocity of *u* and then migrates under the gravity effect.

### 2.2. Numerical Method

The impact of oil-in-air droplets on an aqueous solution is a problem concerning three immiscible fluids, and accurate phase interface tracking is a key problem during simulation. Up to now, there are many methods for tracking fluidic interfaces, such as Lattice Boltzmann, level set, phase field, and volume of fluids [40,41,42]. Among these approaches, the phase field method is attractive because of its better mass conservation, rapid computation speed, and satisfactory algorithm stability [43]. Therefore, in this study, we adopt the three-phase field method to establish a two-dimensional simulation model. To track the fluidic interfaces of three immiscible fluids, the following Cahn–Hilliard equations are solved [44,45]:

(1) $$\frac{\partial \phi_{i}}{\partial t}+\nabla \cdot \left(u\phi_{i}\right)=\nabla \cdot \frac{M_{\phi }}{\Sigma_{i}}\nabla \eta_{i},\;i=w,\;o,\;a$$

(2) $$\eta_{i}=\frac{4\Sigma_{T}}{\epsilon }\underset{j\neq i}{\Sigma }\left(\frac{1}{\Sigma_{j}}\left(\partial_{i}F\left(\phi \right)-\partial_{j}F\left(\phi \right)\right)\right)-\frac{3}{4}\epsilon \Sigma_{i}\nabla^{2}\phi_{i}$$

(3) $$F\left(\phi \right)=\sigma_{wo}\phi_{w}^{2}\phi_{o}^{2}+\sigma_{wa}\phi_{w}^{2}\phi_{a}^{2}+\sigma_{oa}\phi_{o}^{2}\phi_{a}^{2}+\phi_{w}\phi_{Bo}\phi_{a}\left(\Sigma_{w}\phi_{w}+\Sigma_{o}\phi_{o}+\Sigma_{a}\phi_{a}\right)$$

(4) $$\Sigma_{i}=\sigma_{i,j}+\sigma_{i,k}-\sigma_{j,k}$$

(5) $$\frac{3}{\Sigma_{T}}=\frac{1}{\Sigma_{w}}+\frac{1}{\Sigma_{o}}+\frac{1}{\Sigma_{a}}$$

where $\phi_{i}$ is the phase field variable, **u** is the velocity vector, $M_{\phi }$ is the molecular mobility parameter, $\eta_{i}$ is the generalized chemical potential, ϵ is the control parameter of interface thickness, *σ*_*i*,*j*_ is the interfacial tension, and $F\left(\phi \right)$ is the free energy of the three-phase system.

The phase field variables vary between 0 and 1 and are a measure of the concentration of each phase. At each point, the phase field variables satisfy the following equation:

(6) $$\phi_{w}+\phi_{o}+\phi_{a}=1$$

To ascertain continuous variation across the fluidic interface, the density and dynamic viscosity of the fluid mixture are defined as:

(7) $$\rho =\phi_{w}\rho_{w}+\phi_{o}\rho_{o}+\phi_{a}\rho_{a}$$

(8) $$\mu =\phi_{w}\mu_{w}+\phi_{o}\mu_{o}+\phi_{a}\mu_{a}$$

where $\rho_{w}$, $\rho_{o}$, and $\rho_{a}$ are the densities of the aqueous solution, oil, and air, respectively, $\mu_{w}$, $\mu_{o}$ and $\mu_{a}$ are the dynamic viscosities of these three phases, respectively. In our study, the material of the droplet is silicone oil, and the aqueous solution is a mixture of 60 wt% glycerol, 38 wt% deionized water, and 2 wt% polyvinyl alcohol. Their basic physical properties of fluids are listed in Table 1.

The fluids also meet the Navier–Stokes and the continuity equations:

(9) $$\rho \frac{\partial u}{\partial t}+\rho \left(u\cdot \nabla \right)u=\nabla \cdot \left[-pI+\mu \left(\nabla u+{\left(\nabla u\right)}^{T}\right)\right]+\rho g+F_{st}$$

(10) $$\frac{\partial \rho }{\partial t}+\nabla \left(\rho u\right)=0$$

where *ρ* is the volume averaged density, *p* is the pressure, *μ* is the dynamic viscosity of the fluid, *g* is the gravitational acceleration, and ***F****_st_* is the capillary force per unit volume.

The capillary force is described as follows:

(11) $$F_{st}=\underset{i=A,B,C}{\Sigma }\left(\eta_{i}\nabla \phi_{i}\right)$$

### 2.3. Initial and Boundary Conditions

The fluidic phases are initially considered to be quiescent and at uniform pressure,

(12) u=0, and p=constant at t=0

The droplet with an initial velocity of u migrates under gravity. These conditions are the initial conditions employed for the study, whereas the following boundary conditions are employed in order to solve the governing equations.

At the sidewall, the free-slip boundary condition is set,

(13) $$u\cdot n=0,\left(n\cdot \nabla u\right)\times n=0$$

where **n** is the normal vector.

At the bottom wall, we have employed the following no-slip and impermeability conditions:

(14) n×u=0,u·n=0

A pressure point constraint is employed to enforce a zero reference gauge pressure at the central point of the air–aqueous solution interface.

(15) p=0

The boundary conditions for Equation (1) are, in general, homogeneous Neumann type. For the chemical potential $\eta_{i}$, this condition ensures that there is no mass diffusion through the boundary,

(16) $$n\cdot \nabla \eta_{i}=0$$

For the parameter ($\phi_{i}$), wetted wall boundary conditions are incorporated on the side and bottom walls [32],

(17) $$n\cdot \nabla \phi_{i}=\left|\nabla \phi_{i}\right|\cos\left(\theta_{ij}\right)$$

The contact angle of the interface in between phases ‘*i*’ and ‘*j*’ is denoted by $\theta_{ij}$.

### 2.4. Solution Methodology and Validation

**Figure 2:**
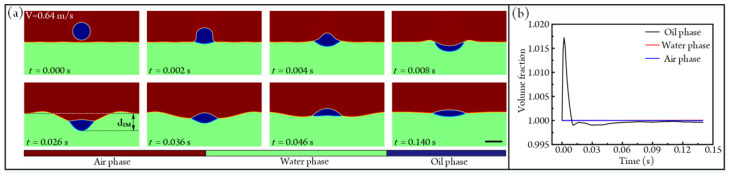
(**a**) The impacting states between an oil droplet and aqueous solution under different time points when the impacting velocity and diameter of the droplet are 0.64 m/s and 4 mm, respectively (see Video S1). Scale bar, 4 mm. (**b**) Volume fractions of three immiscible fluids under different time points.

A commercial software of COMSOL Multiphysics 6.0 is used to solve the aforementioned time-dependent partial differential equations. The variables for flow and phase fields are calculated by using PARDISO solvers. In the simulation, the environmental temperature is fixed at 293.15 K. The droplet, with an initial downward velocity, migrates under the effect of gravity and finally impacts the aqueous solution. Before formal numerical analyses, we conduct a grid dependence test and compare our results with the experiments conducted by Jain et al. [30] to demonstrate the accuracy of the simulation model, as shown in Section S1 of the Supplementary Materials. The suitable agreement between simulations and experiments indicates the accuracy and feasibility of this numerical model. What is more, the simulation model can keep the conservation of mass at different time points, as shown in Figure 2. Specifically, when the impacting velocity of the droplet is 0.64 m/s, the impacting states between the droplet and liquid film under different time points are given in Figure 2a (see Video S1). It can be found that the droplet migrates downward first and is bounced back afterward; finally, it reaches the balanced state at the air–water interface because of the transfer and dissipation of kinetic energy (see Section S2 of the Supplementary Materials). During the impacting process, the volume fractions of the three phases are listed in Figure 2b. Clearly, the mass of the three phases is conservative at different time points, which is also evidence of the feasibility of our numerical model. Thus, the simulation results can help us to understand the droplet-impacting hydrodynamics.

## 3. Results and Discussions

The impacting behaviors between an oil-in-air droplet and an aqueous solutions are influenced by droplet kinetic energy, inertial (gravity) force, interfacial tensions, and viscous force. The magnitudes of these forces are affected by various factors, such as fluid viscosity, density, interfacial tensions, impacting velocity, droplet diameter, and Newtonian and non-Newtonian properties of fluids. To understand their effects on droplet impacting, we will conduct systematic numerical analyses in the following sections.

### 3.1. Influence of Fluid Viscosity on Droplet Impacting

**Figure 3:**
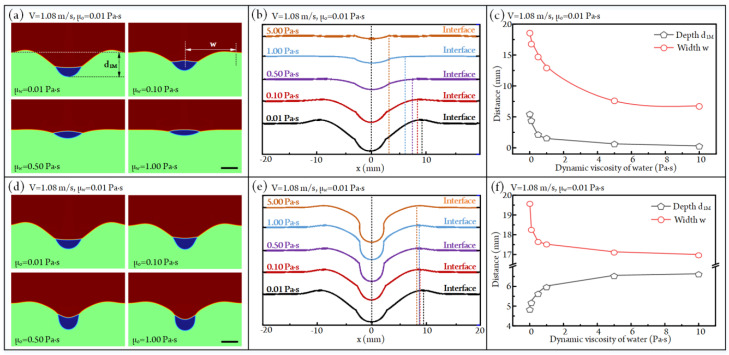
(**a**) The maximum sinking states of the oil droplet after impacting with an aqueous solution under different aqueous solution viscosities at V = 1.08 m/s and μ_o_ = 0.01 Pa·s (see Video S2). Scale bar, 4 mm. (**b**) The configurations of the maximum liquid craters under different aqueous solution viscosities. (**c**) The plots of the maximum crater depth d_1M_ and width w versus dynamic viscosity of aqueous solution at V = 1.08 m/s and μ_o_ = 0.01 Pa·s. (**d**) The maximum sinking states of the oil droplet after impacting with an aqueous solution under different oil viscosities at V = 1.08 m/s and μ_w_ = 0.01 Pa·s (see Video S2). Scale bar, 4 mm. (**e**) The configurations of the maximum liquid craters under different viscosities of droplet oil. (**f**) The plots of the maximum crater depth d_1M_ and width w versus dynamic viscosity of oil at V = 1.08 m/s and μ_o_ = 0.01 Pa·s.

In the droplet (phase o)-aqueous solution (phase w)-air (phase a) system, to analyze the influences of the dynamic viscosities of oil (μ_o_) and aqueous solution (μ_w_) on droplet impacting, the initial downward velocity and diameter of the droplet are fixed at 1 m/s and 4 mm, respectively. Moreover, the impacting velocity will reach V = 1.08 m/s at the moment of impacting under the effect of gravity. Figure 2a indicates that the impacted droplet would migrate downward first and bounce back afterward; finally, it achieves the balanced state at the air–water interface. So, we use the maximum sinking states of the droplet to judge the influences of fluid viscosity on the impacting behaviors between the oil droplet and aqueous solution. The maximum sinking states of oil droplets after impacting with an aqueous solution under different viscosities of aqueous solution are exhibited in Figure 3a (also see Video S2). Clearly, with the increase in aqueous solution viscosity, the sinking depth of the droplet and the size of the water crater both decrease. The configurations of maximum craters under different viscosities of aqueous solution are shown in Figure 3b. Then, we can obtain the quantitative curves of crater sizes versus aqueous solution viscosity, as shown in Figure 3c. It can be found that the crater depth d_1M_ decreases from 18.61 mm to 6.79 mm as the viscosity of the aqueous solution rises from 0.01 Pa·s to 10 Pa·s. As for the width of crater w, it decreases by 93.8% when the viscosity of the aqueous solution is improved from 0.01 Pa·s to 10 Pa·s. This phenomenon is due to the fact that the viscosity of the aqueous solution has a negative effect on fluid flow, resulting in the impact kinetic energy between the droplet and liquid film having a weak effect on droplet sinking and liquid crater formation as the viscosity of the aqueous solution rises.

The maximum sinking states of the oil droplet after impacting with an aqueous solution under different oil viscosities are given in Figure 3d (see Video S2). We can find that the maximum sinking depth of the droplet rises with the rising of oil viscosity. The configurations of the formed liquid crater at this state are shown in Figure 3e. Accordingly, the statistical depth d_1M_ and width w of the liquid crater under different oil viscosities are listed in Figure 3f. It is clear that the crater depth d_1M_ rises from 4.95 mm to 6.64 mm as the oil viscosity rises from 0.01 Pa·s to 10 Pa·s. In contrast, the crater width w is negatively affected by the increased oil viscosity. Specifically, when the oil viscosity increases from 0.01 Pa·s to 10 Pa·s, the w decreases from 19.57 mm to 17.00 mm. This can be explained by the following statements. With the rising of oil viscosity, the deformability of the oil droplet in the horizontal direction decreases during the impacting process, resulting in the impact kinetic energy becoming more concentrated at the impact position. Obviously, the more concentrated impact kinetic energy will lead to an increase in liquid crater depth and a decrease in crater width.

### 3.2. Influence of Fluid Density on Droplet Impacting

**Figure 4:**
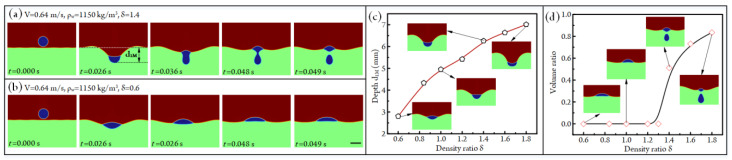
(**a**) Impacting states of the droplet under different time points at V = 0.64 m/s, *ρ*_w_ = 1150 kg/m^3^, and *δ* = 1.4 (see Video S3). (**b**) Impacting states of the droplet under different time points at V = 0.64 m/s, *ρ*_w_ = 1150 kg/m^3^, and *δ* = 0.6 (see Video S3). Scale bar, 4 mm. (**c**) The influence of density ratio *δ* on maximum liquid crater depth d_1M_. (**d**) The ratios of the volume of the droplet entering the aqueous solution to the total volume of the oil droplet under different fluid density ratios.

In the above analyses, the densities of oil and aqueous solution are fixed at 970 kg/m^3^ and 1150 kg/m^3^, respectively. Obviously, the density ratio of oil to water *δ* = *ρ*_o_/*ρ*_w_ will affect the gravity and buoyancy force acting on the droplet. To understand the effect of fluid density on droplet impacting, we fix the density of the aqueous solution at 1150 kg/m^3^ and then adjust the density ratio *δ* to conduct simulations of droplet impacting. When the impacting velocity and density ratio are 0.64 m/s and 1.4, respectively, the impacting states between the oil-in-air droplet and aqueous solution under different time points are shown in Figure 4a (Video S3). After impacting, the droplet first migrates downward until the water crater reaches the maximum size (the impacting state at *t* = 0.026 s). Then, the aqueous solution begins to bounce back, since the upward force of the aqueous solution applied on the droplet is smaller than the gravity of the droplet; the partial oil droplet will pass through the gas–liquid interface and enter the aqueous solution, and the residual oil droplet will be located at the gas–liquid interface. However, at *δ* = 0.6, the total oil droplet will be bounced back because the upward force of the aqueous solution applied on the droplet is higher than the gravity of the droplet, as shown in Figure 4b (Video S3).

The curve of the maximum liquid crater depth d_1M_ versus density ratio is given in Figure 4c. The crater depth d_1M_ is positively affected by the density ratio *δ*. Specifically, d_1M_ rises from 2.8 mm to 7.0 mm when δ is increased from 0.6 to 1.8. During the change of density ratio, the oil droplets in some conditions will be completely bounced back, whereas the partial oil droplet enters an aqueous solution, and the residual one bounces back when the oil density is much higher than that of water. To quantify this influence, we further analyze the ratios of the volume of the droplet entering the aqueous solution to the total volume of the oil droplet under different fluid density ratios, as shown in Figure 4d. It can be found that the bouncing state of the droplet varies when the density ratio *δ* is higher than 1.3. The volume ratio increases from 0 to 0.83 as *δ* rises from 0.6 to 1.8.

### 3.3. Influence of Interfacial Tension on Droplet Impacting

**Figure 5:**
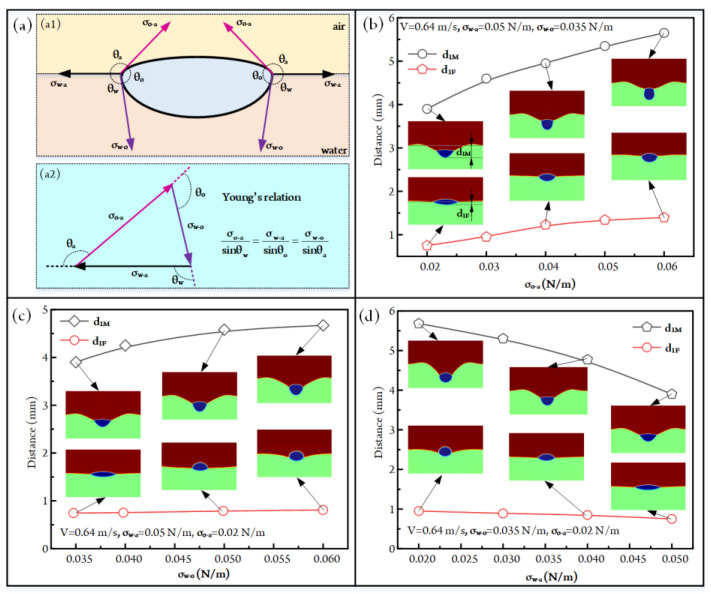
(**a**) The interfacial tensions acting on the droplet when it is in the equilibrium state. (**b**) The influences of σ_o-a_ on the maximum and final depths of the liquid crater at V = 0.64 m/s, σ_w-a_ = 0.050 N/m, and σ_w-o_ = 0.035 N/m (Video S4). d_1M_ signifies the depth of maximum depth of the liquid crater; the size of the liquid crater reaches the highest value when the droplet sinks to the deepest position of water. d_1F_ denotes the depth of the liquid crater when the droplet is in a balanced state. (**c**) The effects of σ_w-o_ on the maximum and final depths of the liquid crater at V = 0.64 m/s, σ_w-a_ = 0.050 N/m, and σ_o-a_ = 0.020 N/m (Video S4). (**d**) The effects of σ_w-a_ on the maximum and final depths of the liquid crater at V = 0.64 m/s, σ_w-o_ = 0.035 N/m, and σ_o-a_ = 0.020 N/m (Video S4).

The configurations of fluidic interfaces are deeply affected by the interfacial tensions of fluids. In order to understand the influence of interfacial tension on droplet-impacting behaviors, we further simulate the impacting hydrodynamics of three phases. The above studies have demonstrated that the droplet would be balanced at the air–liquid interface at the final state. At this stage, the interfacial tension forces acting on the droplet are shown in Figure 5a, and these three interfacial tensions meet Young’s relation. According to Young’s relation, we can conclude that the relative magnitudes of these three interfacial tensions are related to the droplet state at the air–liquid interface.

When conducting simulations, the impacting velocity and droplet diameter are fixed at 0.64 m/s and 4 mm, respectively. The influences of σ_o-a_ on droplet impact and the size of the formed liquid crater are first analyzed, as shown in Figure 5b (see Video S4). It can be seen that the maximum and final depths of the liquid crater are both positively affected by the increased σ_o-a_. Concretely, the maximum crater depth d_1M_ increases from 3.90 mm to 5.65 mm when the σ_o-a_ rises from 0.02 N/m to 0.06 N/m. The final crater depth d_1F_ at the stable state increases by 86.2% as the σ_o-a_ is improved from 0.02 N/m to 0.06 N/m. This is due to the fact that the increased σ_o-a_ makes the improvement of the interfacial free energy, so to maintain the stability of the three-phase fluidic system, more surfaces of the oil droplet will come into contact with the aqueous solution. That is to say, the droplet will have a deeper sinking state, and the crater depth will be higher.

When the impacting velocity, σ_w-a_, and σ_w-o_ are fixed at 0.64 m/s, 0.050 N/m, 0.020 N/m, respectively, we further obtain a series of droplet-impacting states under different σ_w-o_, as exhibited in Figure 5c (also see Video S4). It can be seen that the maximum crater depth d_1M_ rises from 3.90 mm to 4.67 mm as the σ_w-o_ increases from 0.035 N/m to 0.060 N/m. As for the final crater depth d_1F_, it increases by 11.3% when the σ_w-o_ changes from 0.035 N/m to 0.060 N/m. This variation trend can be explained by the following statements. With the rising of σ_w-o_, in order to decrease the interfacial free energy of the fluidic system, the droplet becomes more repellent to contact with an aqueous solution. That is, the contact area between the droplet and the aqueous solution decreases with the increased σ_w-o_. Accordingly, the decreased contact area leads to increased contact stress during impact, resulting in an increase in droplet sinking and liquid crater depth.

Furthermore, the effect of σ_w-a_ on the impacting behaviors between the oil droplet and the aqueous solution is investigated, as illustrated in Figure 5d (see Video S4). With the increase in the liquid–air interfacial tension σ_w-a_, the energy barrier for droplets crossing the gas–liquid interface also increases. Thus, the maximum and final depths of the liquid crater are both negatively affected by the increased σ_w-a_. Specifically, the maximum crater depth d_1M_ reduces from 5.68 mm to 3.90 mm as σ_w-a_ rises from 0.02 N/m to 0.05 N/m. The final crater depth at the stable state decreases by 21% when the σ_w-a_ is increased from 0.02 N/m to 0.05 N/m.

### 3.4. Influence of Impacting Velocity on Droplet Impacting

**Figure 6:**
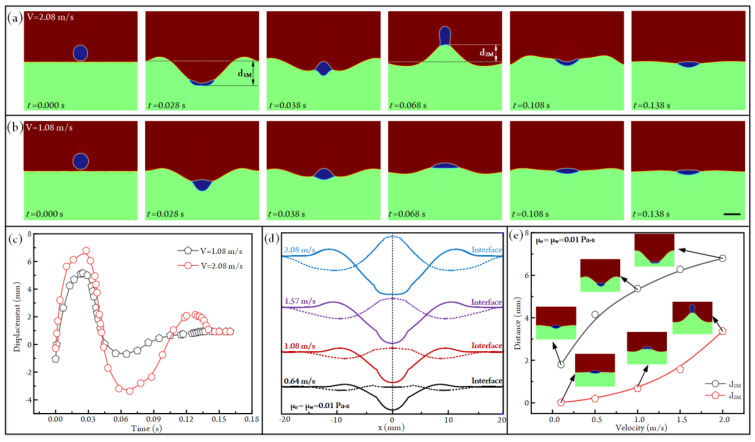
(**a**) Droplet-impacting states under different time points at V = 2.08 m/s and D = 4 mm. d_1M_ signifies the maximum depth of the water crater. d_2F_ denotes the maximum bouncing height of the droplet (Video S5). (**b**) Droplet-impacting states under different time points at V = 1.08 m/s and D = 4 mm. Scale bar, 4 mm (Video S5). (**c**) The plots of the droplet displacement versus time under two different impacting velocities. (**d**) The configurations of air–liquid interfaces when the droplet is in the deepest and highest positions. The solid lines signify that the droplets are in the deepest sinking state, whereas the dashed lines denote that the droplets are in the highest bouncing state. (**e**) The plots of d_1M_ and d_2M_ versus the impacting velocity of the droplet.

The impacting kinetic energy is another factor affecting the impacting behaviors between the oil droplet and the aqueous solution. So, we have set different initial downward velocities for the droplet to conduct simulations. When the impacting velocity and droplet diameter are fixed at 2.08 m/s and 4 mm, respectively, the impacting states of the droplet under different time points are shown in Figure 6a (see Video S5). It is clear that the droplet will migrate downward first and be bounced back afterward; finally, it reaches the balanced state at the air–water interface. During this process, the droplet will have apparent sinking and bouncing distances (d_1M_ and d_2M_). If the impacting velocity is decreased to 1.08 m/s, the sinking and bouncing distances of the droplet and the liquid crater become much smaller, as shown in Figure 6b (Video S5).

To quantify the impacting behaviors of the droplet under different impacting velocities, we have plotted the droplet displacement under different time points in Figure 6c. It is clear that the droplet will experience a sinking-bouncing circle before reaching the equilibrium state at V = 1.08 mm/s. However, when the impacting velocity is increased to 2.08 m/s, the droplet will undergo two sinking-bouncing circles before reaching the equilibrium state, and the sinking and bouncing distances in the second circle are smaller than that in the first circle due to the dissipation of energy. The configurations of the air–liquid interfaces when the crater is in the deepest and highest positions are shown in Figure 6d. Obviously, the impacting velocity has a significant influence on droplet impact. That is, the crater depth and bounced distance of the droplet were both positively affected by the increased impacting velocity. Specifically, the maximum crater depth d_1M_ rises from 1.80 mm to 6.80 mm as the impacting velocity increases from 0.1 m/s to 2 m/s (Figure 6e). Moreover, the maximum bouncing height of the droplet d_2M_ increases from 0.02 mm to 3.37 mm as the impacting velocity is improved from 0.1 m/s to 2 m/s (Figure 6e).

### 3.5. Influence of Droplet Diameter on Impacting States

**Figure 7:**
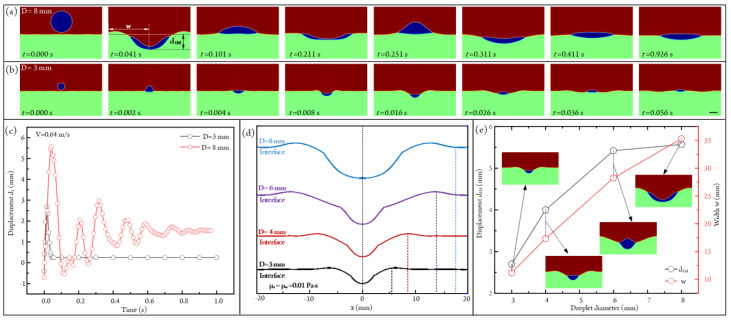
(**a**) Droplet-impacting states under different time points at V = 0.64 m/s and D = 8 mm (Video S6). (**b**) Droplet-impacting states under different time points at V = 0.64 m/s and D = 3 mm (Video S6). Scale bar, 3 mm. (**c**) The plots of the depth of the water crater versus time under two different droplet diameters. (**d**) The configurations of the air–water interfaces under different droplet diameters when the water craters are in the maximum size. (**e**) The plots of maximum water crater depth d_1M_ and crater width w versus droplet diameter.

When two droplets with different sizes have the same velocity, it is obvious that the bigger droplet has the larger kinetic energy compared with that of the smaller one. So the droplet diameter will also influence the impacting behaviors between the oil droplet and the aqueous solution. To understand the specific effect of droplet diameter on droplet impact, the impacting states of droplets with different sizes are simulated, as shown in Figure 7a,b (see Video S6). Apparently, when the impacting velocity is fixed at 0.64 m/s, a bigger liquid crater will form when a larger oil droplet impacts with the aqueous solution. What is more, the droplets with different sizes will experience absolutely different sinking-bouncing circles. According to the curves of droplet displacement versus time plotted in Figure 7c, we can find that the droplet will undergo a sinking-bouncing circle before reaching a stable state when its diameter is 3 mm. In contrast, the drop with a diameter of 8 mm experiences several sinking-bouncing circles before reaching the equilibrium state. This is due to the fact that the droplet with the bigger diameter has a higher kinetic energy. The gradually damped sinking and bouncing of the droplet are related to the transfer and dissipation of kinetic energy.

To quantify the influence of droplet diameter on the impacting behaviors, we have plotted the air–liquid configurations under different droplet diameters when the droplet sinks to the deepest position, as shown in Figure 7d. It is apparent that the maximum crater size rises with the droplet diameter. Specifically, the maximum crater depth d_1M_ increases from 2.70 mm to 5.57 mm as the droplet diameter increases from 3 mm to 8 mm (Figure 7e). As for the crater width w, it improves from 11.17 mm to 35.31 mm when the droplet diameter is increased from 3 mm to 8 mm (Figure 7e).

### 3.6. Influence of Non-Newtonian on Droplet Impacting

In the above analyses, the air, oil, and aqueous solution are all Newtonian fluids; that is, their viscosities keep constant under different shear rates of fluids. Non-Newtonian fluids are common in our daily life, such as water and silicone oil. In contrast, there are also lots of fluids whose viscosities are shear-rate-dependent in the fields of biology, chemistry, and material synthesis, such as human blood and starch solution. After analyzing the Newtonian fluids, we try to further study the droplet behaviors in non-Newtonian fluids. According to the relationship between viscosity and shear rate, non-Newtonian fluids can be classified as many kinds, such as the power-law fluid, Carreau fluid, Bingham fluid, and Casson fluid [49,50,51]. In these non-Newtonian fluids, the power-law fluid is a simple yet typical kind. Many studies concerning non-Newtonian fluids have taken it as an example [52,53]. So, in our study, the viscosities of non-Newtonian oil and aqueous solution also meet the power-law model.

The dynamic viscosity of the power-law fluid is defined by:

(18) $$\mu \left(\dot{\gamma }\right)=K{\dot{\gamma }}^{n-1}$$

where *K* is the consistency coefficient, and its value is fixed at 0.01 Pa·s^n^ in our simulation. *n* is the power-law index. $\dot{\gamma }$ is the rate of the strain tensor, and it can be expressed by the following equation:

(19) $$\dot{\gamma }=\sqrt{\frac{1}{2}{\left(\nabla u+\nabla u^{T}\right)}_{ij}{\left(\nabla u+\nabla u^{T}\right)}_{ji}}$$

The value of power-law index *n* has a significant influence on fluid property:

(20) $$\left\{ \begin{array}{ll} 0<n<1 & \mathrm{shear}\;\mathrm{thinning}\;\mathrm{fluid}\\ n=1 & \mathrm{Newtonian}\;\mathrm{fluid}\\ n>1 & \mathrm{shear}\;\mathrm{thickening}\;\mathrm{fluid} \end{array}\right.$$

**Figure 8:**
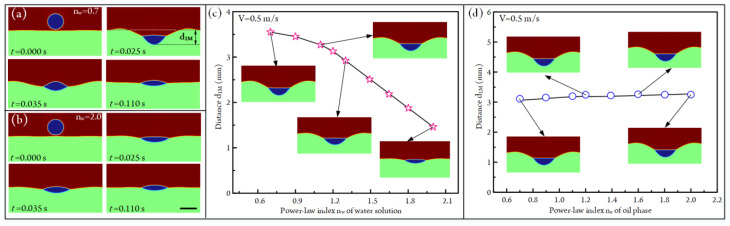
(**a**) Droplet-impacting states under different time points when the power-law index of water and impacting velocity of the droplet are n_w_ = 0.7 and V = 0.5 m/s, respectively (Video S7). (**b**) Droplet-impacting states under different time points when the power-law index of water and impacting velocity of the droplet are n_w_ = 2.0 and V = 0.5 m/s, respectively (Video S7). (**c**) The plot of maximum water crater depth d_1M_ versus power-law index n_w_ of aqueous solution at V = 0.5 m/s. (**d**) The plot of maximum water crater depth d_1M_ versus the power-law index n_o_ of oil at V = 0.5 m/s.

Therefore, we have systematically analyzed the effect of the power-law index on the droplet impact in this section. The influence of the power-law index of aqueous solution n_w_ on droplet impact is first studied. When conducting simulations, the impacting velocity of the droplet is fixed at 0.5 m/s, and the viscosity of the oil is set to be 0.01 Pa·s under different shear rates. The droplet-impacting states under two different n_w_ are portrayed in Figure 8a,b, respectively (also see Video S7). When n_w_ is 0.7, the aqueous solution is a shear-thinning fluid; that is, the shear rate will decrease the viscosity of water. So a large water crater will form under the impact of the droplet (Figure 8a). In contrast, the aqueous solution becomes shear thickening fluid at n_w_ = 2.0; that is, the shear rate will increase fluid viscosity. So the crater size is much smaller compared with that at n_w_ = 0.7 (Figure 8b). The plot of maximum crater depth d_1M_ versus the power-law index n_w_ of water is exhibited in Figure 8c. The d_1M_ decreases from 3.55 mm to 1.46 mm as n_w_ rises from 0.7 to 2. As for the power-law index n_o_ of the oil phase, its effect on crater depth is shown in Figure 8d. It can be seen that the crater depths rise from 3.22 mm to 3.39 mm as n_o_ is improved from 0.7 to 2.0. Obviously, this increasing trend is very small. This is due to the fact that the increased oil viscosity will improve water crater depth (Figure 3f), but n_o_ ranging from 0.7 to 2.0 can only slightly increase the viscosity of the oil phase during impacting.

## 4. Conclusions

In this article, to understand the physics of droplet impacting on another immiscible fluid, we establish a two-dimensional axisymmetric simulation model based on the three-phase field method to conduct numerical analyses. The accuracy of the established numerical model is first validated by the comparison between simulations and experiments. When carrying out simulations, the oil droplet with an initial downward velocity is released into the air phase first. Then, it migrates down under the gravity force, and finally, it impacts the aqueous solution. Under the impact of the oil droplet, a crater will form on the surface of the aqueous solution, which first expands and then collapses with the transfer and dissipation of kinetic energy of this three-phase system. As for the droplet, it flattens, spreads, stretches, or immerses on the crater surface and finally achieves an equilibrium state at the gas–liquid interface after experiencing several sinking-bouncing circles. The simulations indicate that the increased oil viscosity, density, interfacial tension σ_o-a_, σ_w-o_, impacting velocity, and droplet diameter will enhance the droplet penetration depth, water bouncing height, maximum water crater size, and the number of sinking-bouncing circles. In contrast, the impacting effects between droplet and liquid are negatively affected by the improved water viscosity, density, interfacial tension σ_w-a_, and power-law index n_w_. For instance, when the impacting velocity and dynamic viscosity of the aqueous solution are fixed at 1.08 m/s and 0.01 Pa·s, respectively, the maximum crater depth during impacting increases from 4.95 mm to 6.64 mm as the oil viscosity rises from 0.01 Pa·s to 10 Pa·s. These conclusions can help to know about the mechanism of droplet impacting on an immiscible fluid and provide useful guidelines for those applications concerning droplet impacting, such as deep-water oil spills and inkjet printing. Moreover, in the future, we will conduct experiments to analyze the impacting behaviors between oil droplets and aqueous solutions.

## Figures and Tables

**Table 1:** Physical properties of fluids used in this study [46,47,48].

Fluid	Dynamic Viscosity *μ*	Density	Interfacial Tension σ
Silicone oil (phase o)	0.01 Pa·s	970 kg/m^3^	σ_w-o_ = 0.035 N/m
Aqueous solution (phase w)	0.01 Pa·s	1150 kg/m^3^	σ_o-a_ = 0.020 N/m
Air (phase a)	0.0001 Pa·s	1 kg/m^3^	σ_w-a_ = 0.050 N/m

## Data Availability

The data are available as per request.

## Supplementary Materials

The following supporting information can be downloaded at: https://www.mdpi.com/article/10.3390/mi14050951/s1, Figure S1: Grid dependence test at V_0_ = 0.64 m/s, *D* = 4 mm and *t* = 0.1 s; Figure S2:Comparison between simulation and experiments; Video S1: The process of a liquid-in-air droplet impacting on an-other immiscible solution; Video S2: The influence of fluid viscosity on droplet impacting; Video S3: The influence of fluid density on droplet impacting; Video S4: The influence of fluidic interfacial tensions on droplet impacting; Video S5: The influence of impacting velocity on droplet impacting; Video S6: The influence of droplet diameter on droplet impacting; Video S7: The influence of non-Newtonian properties of fluids on droplet impacting.
