# Supplementary material for: Numerical Analysis of Droplet Impacting on an Immiscible Liquid via Three-Phase Field Method
Source: Micromachines (Basel). 2023 Apr 27;14(5):951. doi: 10.3390/mi14050951 (PMC10223795; doi:10.3390/mi14050951)
Supplement: Supplementary file 1 [file micromachines-14-00951-s001.zip › Supplemental Information.pdf]

Supporting information

Numerical Analysis of Droplet Impacting on an Immiscible Liquid via Three-phase Field Method

Qingming Hu [1,2,3,*], Fengshi Hu [1] , Donghui Xu [1] and Kailiang Zhang [4,*]

[1] School of Mechtranoics Engineering, Qiqihar University, Qiqihaer, Heilongjiang, P.R. China 161006; me-huqm@qqhru.edu.cn(Q.H.); 2022908292@qqhru.edu.cn(F.H.); 15946212473@139.com(D.X.)

[2] The Engineering Technology Research Center for Precision Manufacturing Equipment and Industrial Percep-tion of Heilongjiang Province

[3] The Collaborative Innovation Center for Intelligent Manufacturing Equipment Industrialization;

[4] College of Mechanical and Electrical Engineering, Northeast Forestry University, Harbin, Heilongjiang, P.R. China 1500040; e-mail: zkl@nefu.edu.cn, zkl_ljy@163.com

[*] Correspondence: huqm@qqhru.edu.cn(Q.H.); zkl@nefu.edu.cn(K.Z.) Tel.: +86-452-2738241

Electronic Supporting Information (ESI)

Section S1. Validation of the accuracy of simulation model.

Section S2. Energy analysis of droplet impacting

Additional Supplementary Material (AVI):

Video S1: The process of a liquid-in-air droplet impacting on an-other immiscible solution (AVI)

Video S2: The influence of fluid viscosity on droplet impacting (AVI)

Video S3: The influence of fluid density on droplet impacting (AVI)

Video S4: The influence of fluidic interfacial tensions on droplet impacting (AVI)

Video S5: The influence of impacting velocity on droplet impact-ing (AVI)

Video S6: The influence of droplet diameter on droplet impact-ing (AVI)

Video S7: The influence of non-Newtonian properties of fluids on droplet impacting (AVI)

Section S1: Validation of the accuracy of simulation model.

To validate the accuracy of the established simulation model in our study, we conduct the grid dependence test first and compare the simulations with experiments afterwards.

S1.1 Grid dependence test

In simulation, an axisymmetric geometrical model with triangular meshes is established. The grid dependence test is performed when the impacting velocity and droplet diameter are 0.64 m/s and 4 mm repsectively, and the maximum element size ranges from 0.05 mm to 0.35 mm. The plots of the volume fraction of the droplet at t=0.1 s versus mesh size is shown in **Figure S1**. It can be found that the mesh size has a great influence on simulation results before it decreases to the critical value of 0.1 mm. When the mesh size is further smaller than 0.1 mm, although the simulations are accurate, the simulation processes are computationally expensive. Therefore, to balance the calculation speed and the accuracy of simulated results, the triangular mesh with a maximum size of 0.1 mm is finally selected, In the simulation domain, the number of computational elements is about 2.053×10^5. And the time step is 0.001s. During simulation, the program will automatically choose the available time steps according to the physical problem, if the inverse of the time step can gradually decease with the number of simulation iterations and finally keep a small value, the simulation is in the convergence state.

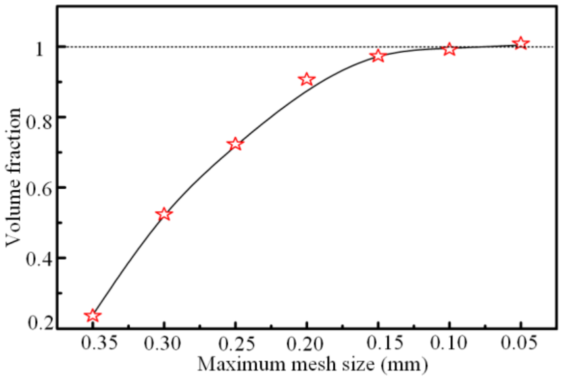

Figure S1. Grid dependence test at V_0=0.64 m/s, D=4 mm and t=0.1s.

S1.2 Comparison between simulation and experiments

After grid dependence test, according to the experimental results in reference [1], we re-establish a similar simulation model and conduct numerical analyses. Then we compare the simulated results with the experiments, as shown in **Figure S2**. It can be seen that the numerical data shows a good agreement with the experiments, proving the accuracy of simulation model in our study. Therefore, the numerical results in our article can give useful guidelines for those applications involving droplet impacting with immiscible solutions.

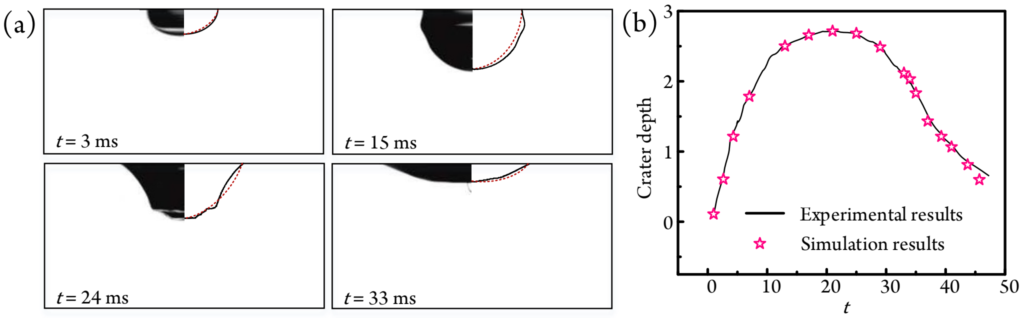

Figure S2. (a) Impacting states between a 35 cSt silicone oil droplet and water solution at Fr = 178, We = 841, Re = 220, corresponding to Ca = 3.82. The black solid lines and the red dashed lines represent the experimental [1] and numerical results respectively. (b) The plots of experimental [1]and numerical dimensionless crater depth versus dimensionless time at Fr = 178, We = 841, Re = 220, corresponding to Ca = 3.82.

Section S2: Energy analysis of droplet impacting.

During the impacting process, the mechanical energy between droplet and water solution will interchangeably transfer, leading to the sinking-bouncing circles of droplets. What's more, the energy will be gradually dissipated by changing into the surface energy and viscous dissipation, resulting in the final stabilization of the oil-air-water system. After impacting, the gravitational energy of water crater (E_P^C), the kinetic energy of crater evolution (E_K^C), the viscous dissipation energy (E_V), the surface energy (E_V) and the remaining kinetic energy of impacting droplet (E_K^D) can be expressed as[2]

$$E_K^C + E_P^C + E_V + E_S = \frac{1}{4} E_K^D \tag{R1}$$

References

[1] U. Jain, M. Jalaal, D. Lohse, and D. van der Meer, Soft Matter 15, 4629 (2019).

[2] LENG, Jong L . Journal of Fluid Mechanics, 2001, 427:73-105.
